# Clinical Indications for Treatment with Multi-Kinase Inhibitors in Patients with Radioiodine-Refractory Differentiated Thyroid Cancer

**DOI:** 10.3390/cancers13092279

**Published:** 2021-05-10

**Authors:** Naoki Fukuda, Shunji Takahashi

**Affiliations:** 1 Department of Medical Oncology, The Cancer Institute Hospital of the Japanese Foundation for Cancer Research, Tokyo 135-8550, Japan; s.takahashi-chemotherapy@jfcr.or.jp; 2 Department of Clinical Cancer Genomics, Hokkaido University Graduate School of Medicine, Sapporo 060-8638, Japan

**Keywords:** differentiated thyroid cancer, multi-kinase inhibitor, lenvatinib, sorafenib

## Abstract

**Simple Summary:**

Differentiated thyroid cancer generally has an indolent, slow-growing nature. The disease gradually progresses in some of the patients and can ultimately develop into life-threatening conditions. Multi-kinase inhibitors including sorafenib and lenvatinib demonstrated prolonged progression-free survival compared with the placebo in pivotal phase 3 trials for patients with radioactive iodine-refractory, progressive disease. However, the overall survival benefit was not confirmed because of the cross-over design of the trials. Therefore, the initiation of treatment with multi-kinase inhibitors should be carefully considered according to the patients and their disease conditions. In this review, we comprehensively describe the currently reported factors that can be potential indications, and further attempt to provide a simplified list of indications for the initiation of multi-kinase inhibitor treatment in patients with radioactive iodine-refractory differentiated thyroid cancer.

**Abstract:**

Differentiated thyroid cancer is usually a slow-growing disease, even if the patients develop distant metastasis. For recurrent or metastatic disease, radioactive iodine therapy is a standard treatment. However, the disease gradually progresses in some of the patients and can ultimately develop into life-threatening conditions. For patients with progressive radioactive iodine-refractory differentiated thyroid cancer (RR-DTC), multi-kinase inhibitors (MKIs) including sorafenib and lenvatinib prolonged progression-free survival compared with placebo in pivotal randomized phase 3 trials, although the benefit in overall survival has not been clearly confirmed, possibly because the patients who received placebo were permitted to cross-over to lenvatinib upon disease progression. Moreover, the adverse events related to MKIs were not negligible. Therefore, the optimal timing of MKI initiation has long been controversial, and physicians should consider various patient and disease factors. Herein, we comprehensively review the clinical factors that can be helpful in determining the initiation of MKIs for patients with RR-DTC.

## 1. Introduction

Thyroid cancer is one of the most common endocrine cancer types [1]. The incidence of thyroid cancer increased five times from 1990 to 2018, though the death rate remained stable at 1–2% [2,3,4,5,6,7]. Indeed, thyroid cancer was the eighth most frequently diagnosed cancer worldwide with 567,000 new cases, whereas cancer-related deaths were estimated to be 41,000 according to the GLOBOCAN 2018 [1,7]. Among all thyroid cancers, more than 95% of cases involve differentiated thyroid cancer (DTC), including papillary thyroid cancer and follicular thyroid cancer [8]. Generally, the natural course of DTC is indolent, with cancer-specific survival rates of 98.6%, 94.7%, and 87.4% at 5, 10, and 15 years, respectively [9]. The incidence of distant metastasis at diagnosis is less than 5% in DTC, although the incidence of recurrence is approximately 15% during follow up, and these recurrent or metastatic diseases can cause thyroid cancer-related death [10,11,12]. Radioactive iodine (I-131) (RAI) is the standard initial therapy for recurrent or metastatic DTC [13,14]. Patients who were initially treated with I-131 and achieved negative imaging studies (negative total body I-131 scans and conventional radiographs) after RAI therapy showed longer survival, with a 10-year survival rate of 92% [15]. However, in some of the patients, the iodine uptake by thyroid cells is diminished and RAI is usually not effective for these patients [10,16,17]. When the patients are refractory to RAI, the 10-year survival rate drops to 10–29% [15].

For patients with RAI-refractory differentiated thyroid cancer (RR-DTC), systemic therapy with multi-kinase inhibitors (MKIs) is a standard option when local treatment options have been exhausted. A summary of two pivotal studies of MKIs for RR-DTC is presented in Table 1. Sorafenib is an oral MKI that inhibits vascular endothelial growth factor receptors (VEGFRs) 1–3, RET, RAF, and platelet-derived growth factor receptor beta (PDGFRβ) signaling [18,19]. The phase 3 DECISION trial demonstrated evidence that sorafenib has benefit in progression-free survival (PFS) in patients with RR-DTC compared with placebo (median PFS 10.8 months vs. 5.8 months; hazard ratio [HR] 0.59, 95% confidence interval [CI] 0.45–0.76, *p* < 0.0001) [20]. Lenvatinib is also an oral MKI targeting VEGFRs 1–3, fibroblast growth factor receptors (FGFRs) 1–4, RET, stem cell factor receptor (KIT), and platelet-derived growth factor receptor alpha (PDGFRα) [21,22,23]. In the phase 3 SELECT trial, lenvatinib demonstrated prolonged PFS compared with placebo in patients with RR-DTC (median PFS 18.3 months vs. 3.6 months, HR 0.21; 99% CI 0.14–0.31, *p* < 0.001) [24]. 

In the National Comprehensive Cancer Network (NCCN) Clinical Practice Guidelines, lenvatinib is recommended as the preferred treatment over sorafenib because of the higher objective response rate reported for lenvatinib (64.8%) compared with sorafenib (12.2%) [20,24,25]. Despite the prognostic significance in PFS, the overall survival (OS) benefit has not clearly been proven compared with placebo for either sorafenib or lenvatinib [20,24]. Therefore, the optimal timing of MKI initiation in patients with indolent and asymptomatic RR-DTC remains controversial. Here, we reviewed the recent findings of factors that can be helpful in making decisions to initiate MKIs, especially lenvatinib, in patients with RR-DTC.

## 2. Controversy over the Optimal Timing for Starting MKIs

In general, systemic chemotherapy should be considered in most solid cancers when recurrent or metastatic lesions become progressive disease. In thyroid cancer, even if radioiodine-refractory, patients with small and slow-growing metastatic lesions are frequently asymptomatic [26]. Patients with asymptomatic indolent disease can temporally enjoy a good quality of life (QOL). Indeed, the incidence of adverse events of sorafenib (98.6%) and lenvatinib (97.3%) is not low [20,24], and the symptoms of certain adverse events, such as fatigue, anorexia, and palmar-plantar erythrodysesthesia syndrome, can directly affect QOL [27,28,29]. Moreover, although MKIs can prolong PFS of RR-DTC patients, they cannot achieve a cure. Because of the cross-over design of the DECISION and SELECT trials, an OS benefit was not demonstrated for sorafenib (HR 0.80, 95% CI 0.54–1.19, *p* = 0.14) or lenvatinib (HR 0.73, 95% CI 0.50–1.07, *p* = 0.1032), compared with placebo [20,24]. Therefore, a “watch and wait” approach has been advocated as an option for patients with asymptomatic indolent RR-DTC [30].

However, the watch and wait approach might increase the risk of disease progression, which can result in invasion of the blood vessels, skin, and mucosa of the bronchi and esophagus. Invasion to these regions can increase the risk of treatment-related fistula bleeding, and some serious and fatal cases related to anti-VEGF treatment have been reported [31]. The watch and wait approach can also increase the risk of symptomatic metastasis. For example, progression of bone metastasis can cause pain and further pathological fracture, which involves pain and worsened activities of daily living [32], and the development of central nervous system (CNS) metastasis can cause CNS symptoms such as paralysis and aphasia [33,34]. The abovementioned disease progression can not only be symptomatic, but can also risk the patients missing the opportunity to receive MKI treatment. Therefore, careful monitoring of disease activity is crucial when the watch and wait approach is selected, and physicians should not miss the optimal timing to start MKIs.

## 3. Recommendation of Each Guideline for Starting MKIs

Several guidelines have mentioned the timing for starting MKIs. The NCCN guidelines recommend that the initiation of MKIs should be considered for patients with rapidly growing lesions or symptomatic disease [25]. The American Thyroid Association (ATA) guidelines state that the administration of MKIs should also be considered when patients have life-threatening lesions (wherein disease progression is expected to require intervention and/or to produce morbidity or mortality in <6 months), in addition to diffuse disease progression and symptomatic disease [35]. In the European Society for Medical Oncology (ESMO) Practice Guidelines, MKIs are recommended for patients with symptomatic disease with multiple lesions or asymptomatic progressive disease with multiple lesions [36]. In addition, patient-related medical factors (age, health status, comorbidities, and contraindications) and patient preferences should be considered with respect to treatment goals and values and acceptance of adverse effects in the European Thyroid Association Guidelines [37]. Similarly, the guidelines of the Japan Association of Endocrine Surgeons indicate that MKIs should not be started unconditionally in all patients with rapid tumor growth or symptoms, but the timing of administration should be determined by considering the benefits and harms associated with the therapy as well as the patient’s general status [38]. As above, there is no strong consensus about when to initiate MKIs for patients with RR-DTC, especially with asymptomatic and progressing disease. Therefore, as a recent review describes, real-world data can assist to optimize the timing of starting MKIs [39].

## 4. Possible Indications for Starting MKIs

Though individual guidelines describe the indications for starting MKIs, the decision is still difficult because of the lack of a clear indication. RIFTOS MKI was a global prospective non-interventional study that investigated the time to symptomatic progression from study entry in asymptomatic patients with progressive RR-DTC [40]. From the final analysis of RIFTOS MKI, the median duration of observation was 27.7 months. The median duration of sorafenib treatment was 13.1 months, which was compatible with the results of the DECISION trial (10.6 months, interquartile range 5.3–15.7 months) [41]. Although the results of RIFTOS MKI demonstrated that 2–3 years of the watch and wait approach is acceptable in some asymptomatic RR-DTC patients, it still cannot provide an indication for starting MKIs at present. Therefore, in clinical practice, physicians should determine the initiation of MKIs using a complex combination of various tumor parameters and the patient’s clinicopathological characteristics. The previously reported clinical factors that can affect the outcomes of MKIs in patients with RR-DTC are summarized in Table 2.

### 4.1. Disease Progression

With regard to the indolent nature of DTC, even if the tumor becomes radioiodine-refractory, MKIs should not always be initiated solely because of the unresectable or metastatic condition of the disease. Indeed, evidence of disease progression within 13–14 months by the Response Evaluation Criteria in Solid Tumors (RECIST) was incorporated in the inclusion criteria of the DECISION and SELECT trials [20,24,56]. Even amongst patients who have progressed within 12 months according to the RECIST, most are asymptomatic, especially those with small tumors (<10 mm). Therefore, conforming to the inclusion criteria of the pivotal trials when patients have RECIST measurable target lesions (lesions with longest diameter ≥10 mm, or lesions with a short axis that measures ≥15 mm for lymph nodes) with progressive disease within 12 months, physicians should consider whether to initiate MKIs [57]. If not, careful active surveillance is required.

### 4.2. Age

According to the Surveillance, Epidemiology, and End Results (SEER) database, the incidence of thyroid cancer peaks at 40–59 years, and the age at diagnosis of thyroid cancer is associated with disease-specific survival [58]. Though the cutoff age is not clear, age is considered as a prognostic factor for thyroid cancer [59].

In the subgroup analysis of the SELECT trial, median OS was not different between the lenvatinib arm and the placebo arm in younger patients (aged ≤65 years) (HR 0.978, 95% CI 0.577–1.656, *p* = 0.933). However, in older patients (aged >65 years), the median OS was significantly longer in the lenvatinib arm than the placebo arm (HR 0.53, 95% CI 0.31–0.91, *p* = 0.02) [42]. Considering the cross-over design of the trial, the results suggest that younger patients can make up for a treatment delay after the watch and wait approach with lenvatinib, whereas older patients cannot, leading to shortened OS. Moreover, the incidence of grade ≥3 adverse events of lenvatinib was significantly higher in older patients than in younger patients (88.7% vs. 67.1%, *p* < 0.001). Dose reductions (73.6% vs. 63.9%), dose interruptions (86.8% vs. 79.4%), and treatment discontinuation (19.0% vs. 11.0%) were also more likely in older patients than younger patients. These results indicate that when the patient is older than 65 years with progressive disease, the initiation of lenvatinib might be considered with careful management of adverse events.

### 4.3. Sex

Sex differences in the incidence and survival of thyroid cancer have been reported. The incidence of papillary thyroid cancer (PTC) among women is almost triple that of men; however, the estimated death rate in women was only 1.3-fold higher than that in men [60,61]. The incidence rate peaked at 40–49 years in women, whereas it peaked at 60–69 years in men [62]. Disease-free survival and mortality was poor in men compared with women in several studies [60,63]. In the DECISION trial, sorafenib showed a benefit in PFS regardless of sex (HR not shown) [20]. In the SELECT trial, prolonged PFS by lenvatinib was observed both in men (HR 0.21, 95% CI 0.14–0.32) and women (HR 0.26, 95% CI 0.16–0.41) compared with the placebo, although the median PFS might be shorter in men (15.1 months) than in women (18.8 months) [24]. These results suggest that the efficacy of MKIs is independent of sex, but the prognosis can be worse in men than in women because of the original unfavorable prognosis in men.

### 4.4. Histological Subtypes

DTC consists of PTC and follicular thyroid cancer (FTC). PTC is the most frequent subtype, which comprises approximately 80% of thyroid cancer, whereas FTC is the second most frequent subtype, accounting for 10–15% of thyroid cancer [63]. FTC is more common in men than women, and the incidence is higher in older individuals compared with PTC [64,65]. The risk of distant metastasis, such as to the lung and bone, is greater in FTC than in PTC, and therefore the overall survival in FTC is reported to be shorter than in PTC [66,67].

In the subgroup analysis of the SELECT trial, lenvatinib demonstrated prolonged PFS both in PTC (HR 0.30, 95% CI 0.20–0.44) and FTC (HR 0.07, 95% CI 0.03–0.21) compared with the placebo [24]. However, an OS benefit was not observed in patients with PTC (HR 0.73, 95% CI 0.50–1.07), whereas OS was significantly longer in patients with FTC (HR 0.41, 95% CI 0.18–0.97) treated with lenvatinib compared with the placebo [68]. This indicated that disease progression is faster in patients with FTC than in patients with PTC. Therefore, a delay in starting lenvatinib can lead to worse OS. For patients with FTC, the initiation of MKIs might be considered earlier than for patients with PTC.

### 4.5. Tumor Location

When tumors are located at certain specific sites, the initiation of MKIs should be considered. Major vessels such as the carotid artery, aortic arch, and pulmonary artery can be at risk of fatal bleeding during anti-VEGF therapy [31]. Lymph node and pulmonary metastases may be adjacent to these major vessels. In patients with metastasis located near major vessels, a watch and wait approach can increase the risk of disease invasion to these major vessels. Similarly, when a metastatic lesion is close to the skin, bronchi, and esophagus, the risk of treatment-related fistula can be increased by a watch and wait approach [69,70]. Patients who previously underwent external beam radiotherapy for these lesions may be at a greater risk of developing an irreversible fistula; therefore, MKIs, especially lenvatinib, should be avoided for these patients [37,69]. The anatomical location should be carefully monitored during active surveillance, and when the lesion is at risk of developing disease progression, MKI initiation should be considered before the lesions invade the major vessels, skin, bronchi, and esophagus.

### 4.6. Metastatic Sites

Metastases from DTC mainly involve the lung, bone, and lymph nodes [71,72]. In the DECISION trial, sorafenib demonstrated prolonged PFS regardless of the presence of bone metastases (HR not shown) [20]. In the SELECT trial, lenvatinib showed a PFS benefit both in patients with (HR 0.21, 95% CI 0.15–0.29) or without (HR 0.24, 95% CI 0.08–0.77) pulmonary metastasis, and in patients with (HR 0.26; 95% CI 0.16–0.42) or without (HR 0.18, 95% CI 0.12–0.27) bone metastasis [24]. In a small phase 2 trial of sorafenib, the median OS was shorter in patients with bone metastasis compared with those without bone metastasis [73]. Moreover, the mean maximum tumor shrinkage with lenvatinib in bone metastasis was smaller (−10.7%) than that in lung (−45.9%), liver (−35.6%), and lymph nodes (−47.5%) in the SELECT trial [74]. The duration of response was also shorter in patients with liver metastasis (15.7 months vs. 30.5 months) and brain metastasis (9.3 months vs. 30.5 months), even in patients who once responded to lenvatinib [43]. Therefore, no definite relationship has been identified between metastatic sites and efficacy of MKIs, though bone, liver, and brain metastases can be less responsive to MKIs. In patients with these metastatic sites, MKIs might be considered earlier than in those with metastases at other sites.

### 4.7. Thyroglobulin and Thyroglobulin Doubling Time

Thyroglobulin (Tg) is a protein produced by follicular cells and is the precursor of the thyroid hormones (thyroxine [T4] and triiodothyronine [T3]) [75]. Serum Tg is the marker for the follow-up of DTC after thyroidectomy [76,77,78]. Several limitations should be considered when using Tg as a marker for thyroid cancer. Tg levels are dependent on serum thyrotropin (TSH) levels, and anti-thyroglobulin antibodies (TgAb) interfere with the measurement of thyroglobulin [79,80].

In the DECISION and SELECT trials, Tg levels decreased during treatment with MKIs, especially in responders, whereas Tg levels did not decrease in patients receiving placebo [20,45]. Similar results were observed in retrospective analyses of real-life clinical practice [81,82]. Sorafenib prolonged PFS compared with placebo, irrespective of high or low Tg levels at baseline (cutoff value 449.4 ng/mL, interaction *p* = 0.992) [20]. Lenvatinib also showed better PFS compared with placebo, regardless of baseline Tg levels (*p* = 0.22) [45]. However, patients with higher baseline Tg levels had significantly worse PFS compared with patients who were treated with placebo in both the DECISION and SELECT trials [44,80]. It is also reported that in PTC patients, higher serum Tg levels is associated with extensive disease [83]. These results indicate that baseline Tg level is not a predictive marker for MKIs but is a significant prognostic factor for RR-DTC. Although the optimal cutoff value has not been established and MKIs are effective regardless of Tg level, MKIs might be considered for patients with high Tg levels due to poor prognosis.

Because serum Tg levels can represent tumor burdens in DTC, the kinetics of Tg levels have also been investigated. It is reported that serum Tg levels after thyroidectomy increased exponentially during follow up, and that the rate of disease progression might be estimated by serial measurement of serum Tg levels [84]. Therefore, a shorter doubling time of Tg can reflect more rapidly growing tumor. Indeed, patients with a shorter (<1 year) thyroglobulin doubling time (Tg-DT) showed a significantly shorter survival time compared with patients with a Tg-DT ≥ 1 year [84]. In several retrospective reports describing the practical use of lenvatinib for RR-DTC, the median Tg-DT was 0.48–0.79 years [49,85,86,87]. This suggests that most patients treated with MKIs in clinical practice have Tg-DT < 1 year; thus, Tg-DT < 1 year may be a possible indication for starting MKIs by serial measurement of Tg levels.

### 4.8. Tumor Volume Doubling Time

Although the RECIST criteria are widely used to evaluate tumor progression, they do not include chronological changes. The RECIST criteria also cannot evaluate three-dimensional tumor volume changes. To evaluate the growth speed of tumors, the tumor volume doubling time (TV-DT) has been proposed. It can be instantly calculated with the Doubling Time, Doubling Rate & Progression Calculator (Kuma Hospital, Hyogo, Japan) as well as the Tg-DT [88]. A previous study showed that patients with a shorter average TV-DT (midDT: defined as the average TV-DT calculated from the tumor dimensions of two index metastatic pulmonary lesions measured in at least 4 consecutive CT scans) (<1 year) had a worse prognosis than patients with a longer midDT [89]. Disease progression within 13–14 months, which was an eligibility criterion of the DECISION and SELECT trials, would be equivalent to approximately 4 years of TV-DT, and disease progression within 6 months would be equivalent to approximately 2 years of TV-DT [90]. In patients with midDT ≤1 year, MKI treatment prolonged midDT up to >3 years in 75% of the patients, whereas midDT was not prolonged in 19% of the patients. Conversely, in patients with midDT >1 year, 97% of the patients could maintain midDT >1 year on MKI treatment. Patients who achieved a prolonged midDT by MKI had significantly better disease-specific survival compared with who did not achieve a prolonged midDT [46]. In another retrospective study, patients with TV-DT ≤ 6 months showed significantly poor PFS compared with patients with TV-DT >5 years (HR 2.70, *p* < 0.01) under sorafenib treatment [47]. It is suggested that MKIs cannot slow the tumor growth speed in certain patients with a midDT ≤1 year, leading to poor survival outcomes. Therefore, during active surveillance, if the TV-DT becomes accelerated and approaches 1 year, MKIs should be considered.

### 4.9. Tumor-Related Symptoms

In a post hoc analysis of the DECISION trial, sorafenib demonstrated prolonged PFS in both asymptomatic (10.8 months vs. 7.2 months, HR 0.60, 95% CI 0.45–0.81) and symptomatic (10.7 months vs. 3.6 months, HR 0.39, 95% CI 0.21–0.72) patients compared with placebo [48]. Several small retrospective analyses of lenvatinib demonstrated that tumor-related symptoms can be negative prognostic factors for both PFS and OS [49]. The existence of tumor-related symptoms can affect patients’ performance status (PS). Most patients enrolled in the large pivotal clinical trials had an Eastern Cooperative Oncology Group (ECOG) PS of 0–1 [20,24], whereas 7.7–15% of the patients had an ECOG PS ≥ 2 in the real-world data of lenvatinib for RR-DTC from several countries [91,92]. ECOG PS (0 vs. ≥1) was a prognostic factor for PFS (HR 0.51, 95% CI 0.34–0.76, *p* = 0.0008) and OS (HR 0.44, 95% CI 0.27–0.73, *p* = 0.001) in a post hoc analysis of the SELECT trial [50]. Taking these results together, tumor-related symptoms can affect patients’ PS, leading to worse survival. Moreover, certain tumor-related symptoms can predict severe adverse events. For example, hemoptysis can be a sign of tumor invasion to the bronchi; thus, patients with repeated hemoptysis can be at high risk of treatment-related bleeding. Therefore, although the evidence is limited, MKIs might ideally be considered before the patients develop tumor-related symptoms during the watch and wait approach. Predicting future symptoms is usually difficult. Careful observation with radiological imaging and evaluation of vital signs can potentially be helpful to detect patients’ symptoms early. For example, patients with pleural dissemination often present pleural effusion which can cause dyspnea. Physicians should carefully evaluate radiological findings to detect even a small amount of pleural effusion, and also check vital signs such as respiration rate, pulse rate, and oxygen saturation. Patient education to contact medical providers when patients recognize signs of symptoms is also essential.

### 4.10. Tumor Burden

Tumor size has been postulated as a prognostic factor for thyroid cancer in several reports [93,94]. Larger tumor volume is associated with poor blood supply and elevated interstitial pressure, leading to a hypoxic microenvironment in the tumor [95,96]. Tumor hypoxia may be involved in drug resistance to anti-VEGF therapy including MKIs [97,98,99]. The median sum of the target lesions was 71 mm in the DECISION trial and 59.1 mm in the SELECT trial [20,74]. Both sorafenib and lenvatinib demonstrated preferable efficacy compared with placebo, regardless of tumor size [20,24]. In the post hoc analysis of the SELECT trial results, the cutoff value of the baseline tumor size was selected as 40 mm by receiver operating characteristic analysis, and the patients with larger tumors (>40 mm) had significantly poor OS compared with the patients with small tumors (≤40 mm) (29.1 months vs. not reached, HR 0.42, 95% CI 0.28–0.63) in the lenvatinib arm [51]. Similar results were reported in a retrospective analysis using a baseline tumor size cutoff value of 42 mm [52].

In another post hoc analysis of the SELECT trial, the median OS of patients with any size of lung metastasis was not significantly different between the lenvatinib arm and the placebo arm (43.2 months vs. 34.0 months, HR 0.76, 95% CI 0.57–1.01, *p* = 0.0549), whereas for patients with ≥10 mm lung metastasis, median OS was longer in the lenvatinib arm compared with the placebo arm (44.7 months vs. 33.1 months, HR 0.63, 95% CI 0.47–0.85, *p* = 0.0025) [53].

These results indicate that when lung metastases progress up to >10 mm, a treatment delay can affect the whole survival time, and when the sum of the target lesions is up to approximately 40 mm, then initiation of MKIs should be considered.

### 4.11. Neutrophil-to-Lymphocyte Ratio

Cancer-related inflammation and host immune response have been shown to have crucial roles in tumor progression in several cancer types [100]. The neutrophil-to-lymphocyte ratio (NLR) is calculated by dividing the absolute neutrophil count by the absolute lymphocyte count with complete blood cell counts. It can reflect the balance of the immune system and was associated with survival in solid tumors in a systematic review and meta-analysis [101]. An elevated NLR was associated with extrathyroidal invasion, bilateral, multifocal, and lymph node-positive disease, and with poor prognosis in PTC [102]. The NLR was also significantly elevated in anaplastic thyroid cancer (ATC) patients compared with PTC patients, and can therefore be used as a diagnostic marker to discriminate between ATC and DTC [103].

In the Japanese phase 2 trial of lenvatinib for thyroid cancer including DTC, medullary thyroid cancer (MTC), and ATC, higher pre-treatment NLR was correlated with a shorter PFS following lenvatinib treatment (HR 1.07, 95% CI 1.00–1.14, *p* = 0.06) [104]. In a small retrospective analysis, RR-DTC patients with an elevated NLR (≥3) at baseline showed shorter OS compared with patients with a lower NLR (<3) treated with lenvatinib (11.9 months vs. 35.0 months, *p* < 0.05) [54]. In the post hoc analysis of the SELECT trial, patients with a lower NLR (<3) demonstrated better PFS (HR 0.43, 95% CI 0.29–0.65, *p* < 0.0001) and OS (HR 0.48, 95% CI 0.29–0.78, *p* = 0.0029) compared with those with a higher NLR (≥3) [55]. The NLR value was also demonstrated to reflect the tumor aggressiveness before and during lenvatinib treatment; thus, NLR can be a tumor marker for RR-DTC treated with lenvatinib during active surveillance [54]. An NLR value of 3 is a possible indication for lenvatinib initiation.

### 4.12. Genetic Landscape

Recent advances in precision medicine revealed that thyroid cancers harbor several specific genetic alterations. PTC is associated with *BRAF* mutations (29–83%) [105,106,107], *RET* (RET proto-oncogene) rearrangements (14–43%) [108,109,110,111], *RAS* mutations (0–21%) [112,113], and *NTRK* (neurotrophic tyrosine kinase receptor) rearrangements (3–13%) [108,114]. FTC is also associated with *RAS* mutations (40–53%) [112,115]. Therefore, post hoc analyses according to genetic alterations were undertaken in the DECISION and SELECT trials.

Both sorafenib and lenvatinib demonstrated a PFS benefit regardless of *BRAF* and *RAS* status compared with the placebo [20,45]. However, in the DECISION trial, patients with *BRAF* mutations showed longer PFS than patients without *BRAF* mutations in the placebo arm (HR 0.51, 95% CI 0.32–0.83, *p* = 0.006). Conversely, *RAS* mutations were associated with worse PFS compared with wild-type *RAS* (HR 1.80, 95% CI 1.08–2.99, *p* = 0.022) [44]. Similar results were observed in the SELECT trial in which wild-type *BRAF* was a significant prognostic factor for worse PFS in the placebo arm (*p* = 0.0083) [45]. These results suggested that a treatment delay can affect entire survival outcomes in patients with wild-type *BRAF* RR-DTC, though the approval of genetic testing varies by country.

## 5. Perspectives in the Era of Specific Treatment

Advances in the understanding of the molecular pathways and genetic landscape involved in thyroid cancer have improved clinical outcomes in the treatment of patients with RR-DTC. Sorafenib and lenvatinib are approved and used in daily clinical practice in various countries, and further specific treatment such as with BRAF [116], RET [117], NTRK inhibitors [118] demonstrated favorable results in patients with the respective genetic alterations. These mutation-specific treatments have been approved in several countries. Although the risk of off-target adverse events by the mutation-specific treatments can be reduced because of the highly selective inhibition of the pathway, several notable adverse events can occur by these treatments. For example, cardiac toxicities, ocular toxicities, and cutaneous malignancies by BRAF/MEK inhibition [119]. In general, placebo-controlled trials for these new drugs are unlikely to be conducted, and standard approved treatment is usually selected as a control. For example, a phase 3 trial comparing selpercatinib against standard treatment (cabozantinib or vandetanib) for medullary thyroid cancer is currently in progress [120]. Because the mutation-specific drugs are currently approved in several countries according to the results of phase 2 trials for DTC [116,117,118], it is difficult to evaluate the benefit in OS of these treatments compared with watch and wait, especially for patients with slow-growing disease. Therefore, even if these mutation-specific treatments were to be approved worldwide, physicians should still consider the risk and benefit when starting the treatment. The accumulation of real-world data will be required to modify treatment indications for these specific treatment in the future.

## 6. Conclusions

**Figure 1:**
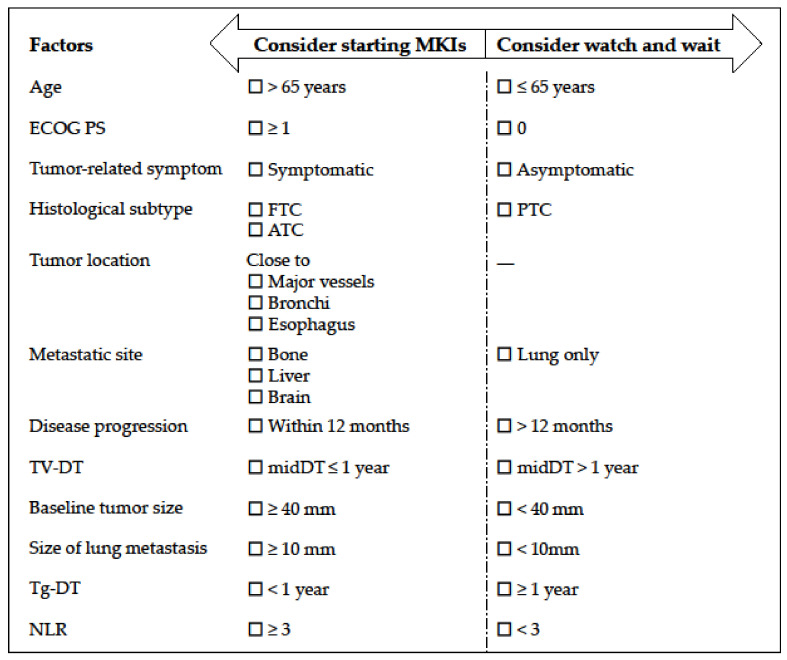
A simple check list of factors which can be potential indications for starting multi-kinase inhibitor treatment for radioactive-iodine refractory differentiated thyroid cancer. ECOG PS, Eastern Cooperative Oncology Group performance status; FTC, follicular thyroid cancer; ATC, anaplastic thyroid cancer; PTC, papillary thyroid cancer; TV-DT, tumor volume doubling time; midDT, average tumor volume doubling time; Tg-DT, thyroglobulin doubling time; NLR, neutrophil-to-lymphocyte ratio.

At present, physicians must determine the initiation of MKIs not only by single factors, but by considering a combination of various clinical parameters, most of which were identified in post hoc or retrospective analyses. Figure 1 describes a simplified check list of clinical indications for starting MKIs according to the real-world data. The obvious limitation of the present review is that we could not evaluate the significance of each factor to the decision making. A Quantitative scale such as a risk scoring system or nomograms is required, but a large-scale prospective cohort with a longer follow-up time is necessary to establish these systems. Future studies are necessary to investigate more effective methods of determining the optimal indications and timing for systemic therapies for RR-DTC.

## Figures and Tables

**Table 1:** Summary of the pivotal phase 3 clinical trials of MKIs in patients with RR-DTC.

Trial Name	Decision [20]	Select [24]
Study design	Randomized controlled trial	Randomized controlled trial
No. of patients	417 (Sorafenib 207, placebo 210)	392 (Lenvatinib 261, placebo 131)
Eligibility criteria	Age ≥ 18 years Locally advanced or metastatic DTC Progression within 14 months RAI-refractory TSH < 0.5 mIU/L	Age ≥ 18 years Locally advanced or metastatic DTC Progression within 13 months RAI-refractory
Experimental arm	Sorafenib	Lenvatinib
Control arm	Placebo	Placebo
Primary endpoint	Progression-free survival	Progression-free survival
Patients’ characteristics	Male 52%, Female 48% PTC 57%, FTC 25%, PDTC 10%, others 9% Metastasis to lung 86%, LN 51%, bone 27%, liver 14%	Male 77%, Female 23% PTC 51%, FTC 37%, PDTC 12% Metastasis to lung 89%, bone 39%
Median PFS	10.6 months vs. 5.8 months HR 0.59, 95% CI 0.45–0.76, *p* < 0.0001	18.3 months vs. 3.6 months HR 0.21; 99% CI 0.14–0.31, *p* < 0.001
Overall response rate	12.2% vs. 0.5%, *p* < 0.0001	64.8% vs. 1.5%, *p* < 0.001
Overall survival	HR 0.80, 95% CI 0.54–1.19, *p* = 0.14	HR 0.73, 95% CI 0.50–1.07, *p* = 0.10

MKI, multi-kinase inhibitor; RR-DTC, radioactive-iodine refractory thyroid cancer; DTC, differentiated thyroid cancer; RAI, radioactive iodine; TSH, thyroid-stimulating hormone; PTC, papillary thyroid cancer; FTC, follicular thyroid cancer; PDAC, poorly differentiated thyroid cancer; LN, lymph node; PFS, progression-free survival; HR, hazard ratio; CI, confidence interval.

**Table 2:** Summary of reported clinical factors that affect outcomes of MKIs in patients with RR-DTC.

Parameter	Agent	Study Design	Results	Reference
Age	Lenvatinib	Prespecified subanalysis	Outcomes: OS (LEN vs. Pbo) Age ≤65 years: HR 0.98, 95% CI 0.58–1.66, *p* = 0.90 Age >65 years: HR 0.53, 95% CI 0.53–0.91, *p* = 0.01	[42]
Sex	Lenvatinib	Prespecified subanalysis	Outcomes: PFS (LEN vs. Pbo) Male: 15.1 months vs. 3.5 months (HR 0.21, 95% CI 0.14–0.32) Female: 18.8 months vs. 3.7 months (HR 0.26, 95% CI 0.16–0.41)	[24]
Histological subtype	Lenvatinib	Prespecified subanalysis	Outcomes: OS (LEN vs. Pbo) Papillary: HR 0.73, 95% CI 0.50–1.07 Follicular: HR 0.41, 95% CI 0.18–0.97	[24]
Liver metastasis	Lenvatinib	Prespecified subanalysis	Outcomes: Duration of response Liver metastasis (+) vs. (−): 15.7 months vs. 30.5 months	[43]
Brain metastasis	Lenvatinib	Prespecified subanalysis	Outcomes: Duration of response Brain metastasis (+) vs. (−): 9.3 months vs. 30.5 months	[43]
Baseline Tg level	Sorafenib	Post hoc	Outcomes: PFS (Tg high vs. Tg low) HR 2.03, 95% CI 1.52–2.71, *p* < 0.001 (Cut-off point: 1021 ng/mL)	[44]
Baseline Tg level	Lenvatinib	Exploratory analysis	Outcomes: PFS (Tg high vs. Tg low) Univariate *p* = 0.027, Multivariate *p* = 0.051 (Cut-off point: 1st quartile)	[45]
Tumor volume doubling time	Lenvatinib	Retrospective	Outcomes: MST Achieved midDT ≥ 3 years: 7.1 years Achieved midDT 1–3 years: 5.6 years Achieved midDT ≤ 1 year: 2.8 years	[46]
Tumor volume doubling time	Sorafenib	Retrospective	Outcomes: PFS TV-DT ≤ 6 months: HR 2.70, 95% CI 1.33–5.45 TV-DT 6 months–1 year: HR 2.06, 95% CI 0.92–4.63 TV-DT 1–5 years: HR 1.35, 95% CI 0.69–2.63 TV-DT >5 years: Reference	[47]
Tumor-related symptoms	Sorafenib	Post hoc	Outcomes: PFS (SOR vs. Pbo) Asymptomatic: 10.8 months vs. 7.2 months (HR 0.60, 95% CI 0.45–0.81) Symptomatic: 10.7 months vs. 3.6 months (HR 0.39, 95% CI 0.21–0.72)	[48]
Tumor-related symptoms	Lenvatinib	Retrospective	Outcomes: PFS Symptomatic vs. asymptomatic: Relative risk 111.8, 95% CI 7.90–1581.3, *p* < 0.01	[49]
ECOG PS	Lenvatinib	Post hoc	PS 0 vs. ≥1: Outcomes: PFS HR 0.51, 95% CI 0.34–0.76, *p* = 0.0008 Outcomes: OS HR 0.44, 95% CI 0.27–0.73, *p* = 0.001	[50]
Baseline tumor size	Lenvatinib	Post hoc	Outcomes: OS Baseline tumor size >40 mm vs. ≤40 mm: 29.1 months vs. NR, HR 0.42, 95% CI 0.28–0.63	[51]
Baseline tumor size	Lenvatinib	Retrospective	Baseline tumor size ≤42 mm vs. >42 mm Outcomes: PFS HR 3.37, 95% CI 1.26–9.02, *p* < 0.02 Outcomes: OS HR 4.14, 95% CI 1.42–12.11, *p* < 0.01	[52]
Size of lung metastasis	Lenvatinib	Post hoc	Outcomes: OS (LEN vs. Pbo) Lung metastasis (any): 43.2 months vs. 34.0 months (HR 0.76, 95% CI 0.57–1.01, *p* = 0.0549) Lung metastasis (≥10 mm): 44.7 months vs. 33.1 months (HR 0.63, 95% CI 0.47–0.85, *p* = 0.0025)	[53]
Neutrophil-to-lymphocyte ratio	Lenvatinib	Retrospective	Outcomes: OS NLR ≤ 3 vs. NLR > 3: 35.0 months vs. 11.9 months (*p* < 0.05)	[54]
Neutrophil-to-lymphocyte ratio	Lenvatinib	Post hoc	NLR ≤ 3 vs. NLR > 3 Outcomes: PFS HR 0.43, 95% CI 0.29–0.65, *p* < 0.0001 Outcomes: OS HR 0.48, 95% CI 0.29–0.78, *p* = 0.0029	[55]
*BRAF* mutation	Sorafenib	Exploratory analysis	Outcomes: PFS *BRAF*-mutant vs. *BRAF*-wild HR 0.51, 95% CI 0.32–0.83, *p* = 0.006	[44]
*BRAF* mutation	Lenvatinib	Exploratory analysis	Outcomes: PFS *BRAF*-mutant vs. *BRAF*-wild HR not shown, *p* = 0.0083	[45]

MKI, multi-kinase inhibitor; RR-DTC, radioactive-iodine refractory thyroid cancer; OS, overall survival; LEN, lenvatinib; Pbo, placebo; HR, hazard ratio; CI, confidence interval; Tg, thyroglobulin; MST, median survival time; midDT, average tumor volume doubling time; SOR, sorafenib; TV-DT, tumor-volume doubling time; ECOG PS, Eastern Cooperative Oncology Group performance status; NLR, neutrophil-to-lymphocyte ratio.
